# Engineering pericyte-supported microvascular capillaries in cell-laden hydrogels using stem cells from the bone marrow, dental pulp and dental apical papilla

**DOI:** 10.1038/s41598-020-78176-7

**Published:** 2020-12-09

**Authors:** S. Prakash Parthiban, Wenting He, Nelson Monteiro, Avathamsa Athirasala, Cristiane Miranda França, Luiz E. Bertassoni

**Affiliations:** 1 Division of Biomaterials and Biomechanics, Department of Restorative Dentistry, School of Dentistry, Oregon Health & Science University, Portland, OR USA; 2 Department of Biomedical Engineering, School of Medicine, Oregon Health & Science University, Portland, OR USA; 3 Cancer Early Detection Advanced Research (CEDAR) Center, Knight Cancer Institute, Oregon Health & Science University, Portland, OR USA; 4 Center for Regenerative Medicine, School of Medicine, Oregon Health & Science University, Portland, OR USA

**Keywords:** Biotechnology, Tissue engineering

## Abstract

Engineered tissue constructs require the fabrication of highly perfusable and mature vascular networks for effective repair and regeneration. In tissue engineering, stem cells are widely employed to create mature vascularized tissues in vitro. Pericytes are key to the maturity of these vascular networks, and therefore the ability of stem cells to differentiate into pericyte-like lineages should be understood. To date, there is limited information regarding the ability of stem cells from the different tissue sources to differentiate into pericytes and form microvascular capillaries in vitro. Therefore, here we tested the ability of the stem cells derived from bone marrow (BMSC), dental pulp (DPSC) and dental apical papilla (SCAP) to engineer pericyte-supported vascular capillaries when encapsulated along with human umbilical vein endothelial cells (HUVECs) in gelatin methacrylate (GelMA) hydrogel. Our results show that the pericyte differentiation capacity of BMSC was greater with high expression of α-SMA and NG2 positive cells. DPSC had α-SMA positive cells but showed very few NG2 positive cells. Further, SCAP cells were positive for α-SMA while they completely lacked NG2 positive cells. We found the pericyte differentiation ability of these stem cells to be different, and this significantly affected the vasculogenic ability and quality of the vessel networks. In summary, we conclude that, among stem cells from different craniofacial regions, BMSCs appear more suitable for engineering of mature vascularized networks than DPSCs or SCAPs.

## Introduction

The human vasculature is a vital component of any cellularized tissue in the body. Blood vessels and capillaries supply all cells with oxygen, nutrients, and paracrine signals for efficient tissue growth and homeostasis. Therefore, a critical hallmark of any engineered tissue is that, after implantation, it should be supplied with sufficient blood to avoid ischemia, hypoxia, and necrosis^1^. Vascular tissue engineering addresses these problems by facilitating the fabrication of engineered tissues with a functional vascular system that is able to replicate the circulatory and biological role of the host vasculature^2–5^. However, understanding and controlling the complex heterotypic interactions that are required to establish a functional vasculature in engineered tissues remains a challenge. Much has been learned about the process of endothelial cell morphogenesis during angiogenesis and vasculogenesis, both during tissue development and remodeling^5–8^. However, control over the myriad interactions occurring between endothelial cells and other mural cells that contribute to the formation of functional vascular capillaries remains poorly understood.

One of the key components of the native vasculature are perivascular mural cells that surround the endothelial lining of the peripheral microvasculature^9–11^. Endothelial cells and pericytes complement each other during  blood vessel formation, with endothelial cells lining the interior of the tubule wall, whereas pericytes cover the exterior of the vascular tube providing vessel stabilization, barrier function, regulation of blood flow, and immune regulation^12–18^. Typically, pericytes are surrounded by a basement membrane, and present an archetypal morphology ranging from spindle to stellate across the microvascular tree^19^. Functional vascular capillaries stabilize pericytes by maintaining the integrity of cell–cell junction and synthesis of the extracellular matrix (ECM) in the basement membrane. Therefore, these cells are largely recognized for being necessary for proper functioning of the small blood vessels^15^. In the absence of pericytes, a dysfunctional microvasculature is established, presenting hemorrhagic and hyper dilated capillaries that can lead to conditions such as hypertension, diabetes, edema, and embryonic lethality^20^. Therefore, the establishment of functional pericytes into vascularized biomaterials is a desirable step for repair and regeneration of highly cellularized tissues.

It has long been known that pericytes derive from the differentiation of stem cells from the mesenchyme in various tissues^21^. However, the inherent ability of stem cells from different sources to differentiate into pericytes during the formation of vascular capillaries remains poorly understood, especially in the context of craniofacial and dental regeneration, where repair and healing often involves tissues and structures that are inherently highly vascularized, such as the skeletal muscle, bone and the dental pulp. Several reports have demonstrated the ability of stem cells from different origins to differentiate into pericytes^22–24^. In fact, a recent systematic review evaluated 20 publications reporting on the differentiation of stem cells into pericytes, and concluded that these cells can serve as a potential source of pericytes with varying potential. However, there is limited information available in the literature to directly compare stem cells from different tissue sources with respect to their ability to form pericyte-supported vascular capillaries for tissue engineering^25^. To address this limitation, here we compared stem cells isolated from different craniofacial tissues, and analyzed their ability to differentiate into pericytes and to form microvascular capillaries, both when seeded in two-dimensional (2D) substrates, as well as embedded in three-dimensional (3D) cell-laden biomaterials. We hypothesized that stem cells from different craniofacial regions would result in significantly different vasculogenic patterns and pericyte-differentiation ability. Due to their ease of availability and sourcing as well as their ubiquity in cell based regenerative applications, we chose to study bone marrow mesenchymal stem cells (BMSC) and investigate their potential to differentiate into pericyte like cells^26^. We compared their behavior with those of two other stem cell types that can be easily harvested from healthy extracted teeth, such as third molars, namely stem cells from the apical papilla (SCAP) and dental pulp stem cells (DPSC). These represent easily accessible and expandable stem cell sources, with proven regenerative potential, thereby making them ideal candidates for tissue engineering applications^27,28^. To that end, we cultured these stem cells either individually or in co-culture with human umbilical vein endothelial cells (HUVEC) in gelatin methacrylate (GelMA) hydrogels. Our results demonstrate that the ability of stem cells to differentiate into a perivascular lineage when co-cultured with HUVECs is significantly distinct depending on the tissue source. Moreover, stem cells from different tissue types show a significant difference of trophic effects on the vasculogenic ability of endothelial cells in cell-laden hydrogels.

## Results

**Figure 1:**
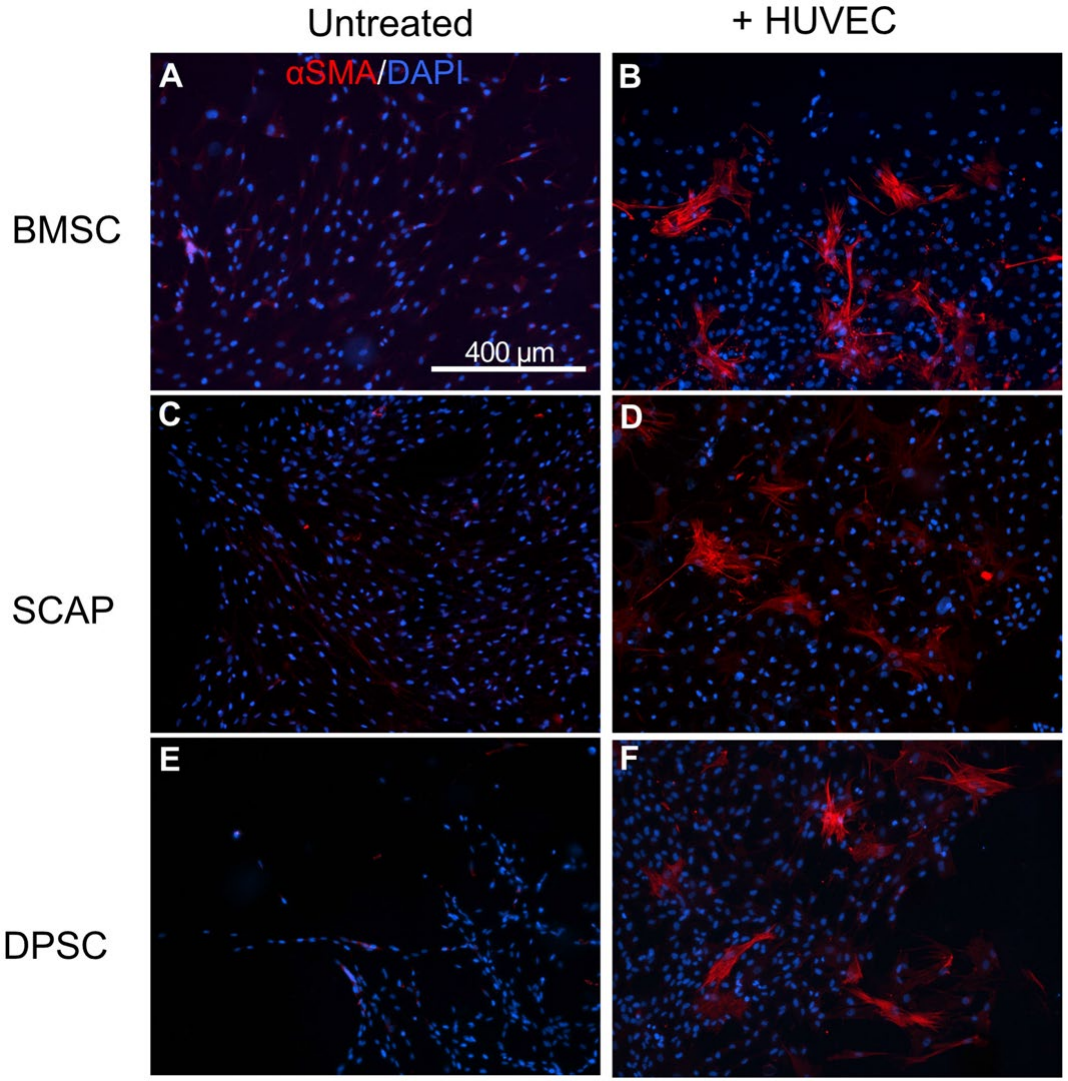
(**A**, **C**, **E**) Stem cells seeded onto GelMA hydrogels (2D) without HUVECs showed no expression of α SMA. (**B**, **D**, **F**) When co-cultured with HUVECs, stem cells from all three sources showed visible expression of the pericyte-related marker α-SMA, thus suggesting their consistent differentiation.

**Figure 2:**
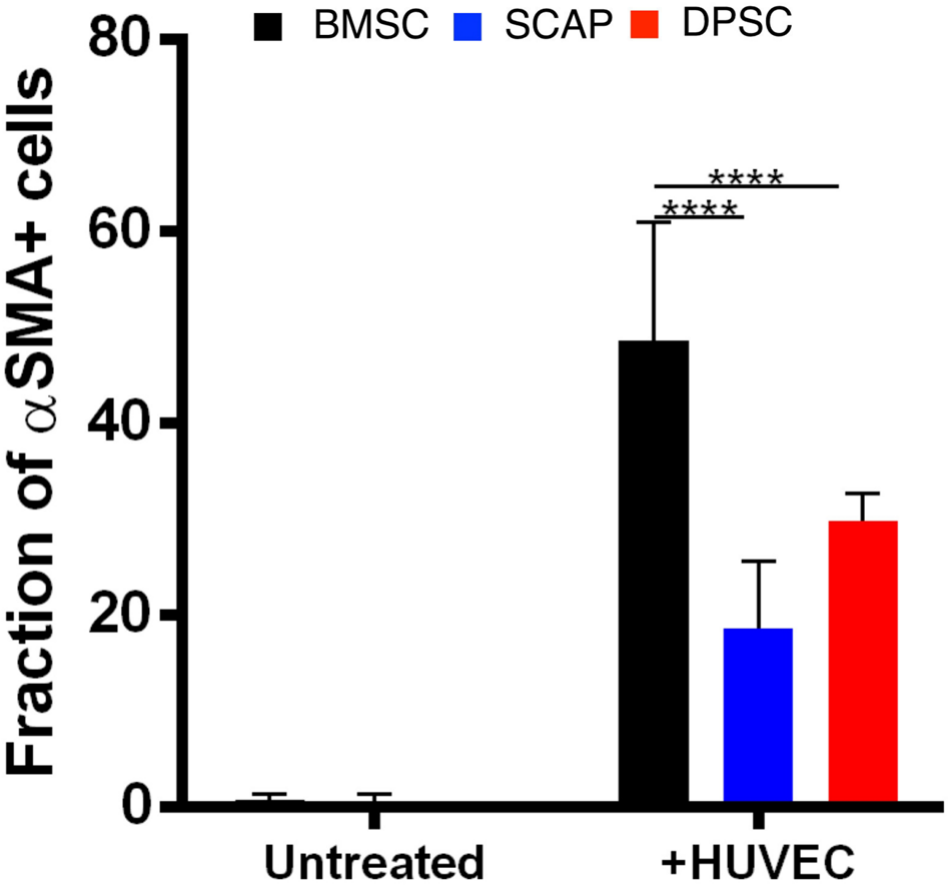
Quantification of α-SMA positive cells on GelMA hydrogels. The untreated groups are the monocultured stem cells without HUVECs, i.e., the BMSC, SCAP and DPSC. The untreated groups hardly expressed any α SMA positive cells. However, when cocultured with HUVECs, the stem cells differentiated to express α-SMA. The number of α-SMA positive cells was higher in BMSC system followed by DPSC and SCAP cells. (****p < 0.0001).

Figure 1 shows stem cells from bone marrow (BMSC), apical papilla (SCAP) and dental pulp (DPSC), either alone or in co-culture with HUVECs, on 2D GelMA hydrogels. For 2D experiments, we chose to use the late stage marker, α-SMA for pericyte identification. All the cells did not show pericyte differentiation when cultured alone on GelMA hydrogel, while co-culture with HUVECs, all groups showed a marked differentiation potential among the stem cells. BMSCs had a better differentiation ability than the other stem cells. The morphology of the differentiated cells in all the stem cell systems looked nearly identical, with some cells showing extended processes, bearing in mind that the 2D substrate affect the way that cells spread and communicate with endothelial cells. The fraction of α-SMA positive cells with BMSC was 2.5 times higher when compared to SCAP (*p* < 0.0001), and 1.6 times higher compared to DPSC (*p* < 0.0001) (Fig. 2). Next, we evaluated the behavior of stem cells in 3D by encapsulating them in GelMA hydrogels.

**Figure 3:**
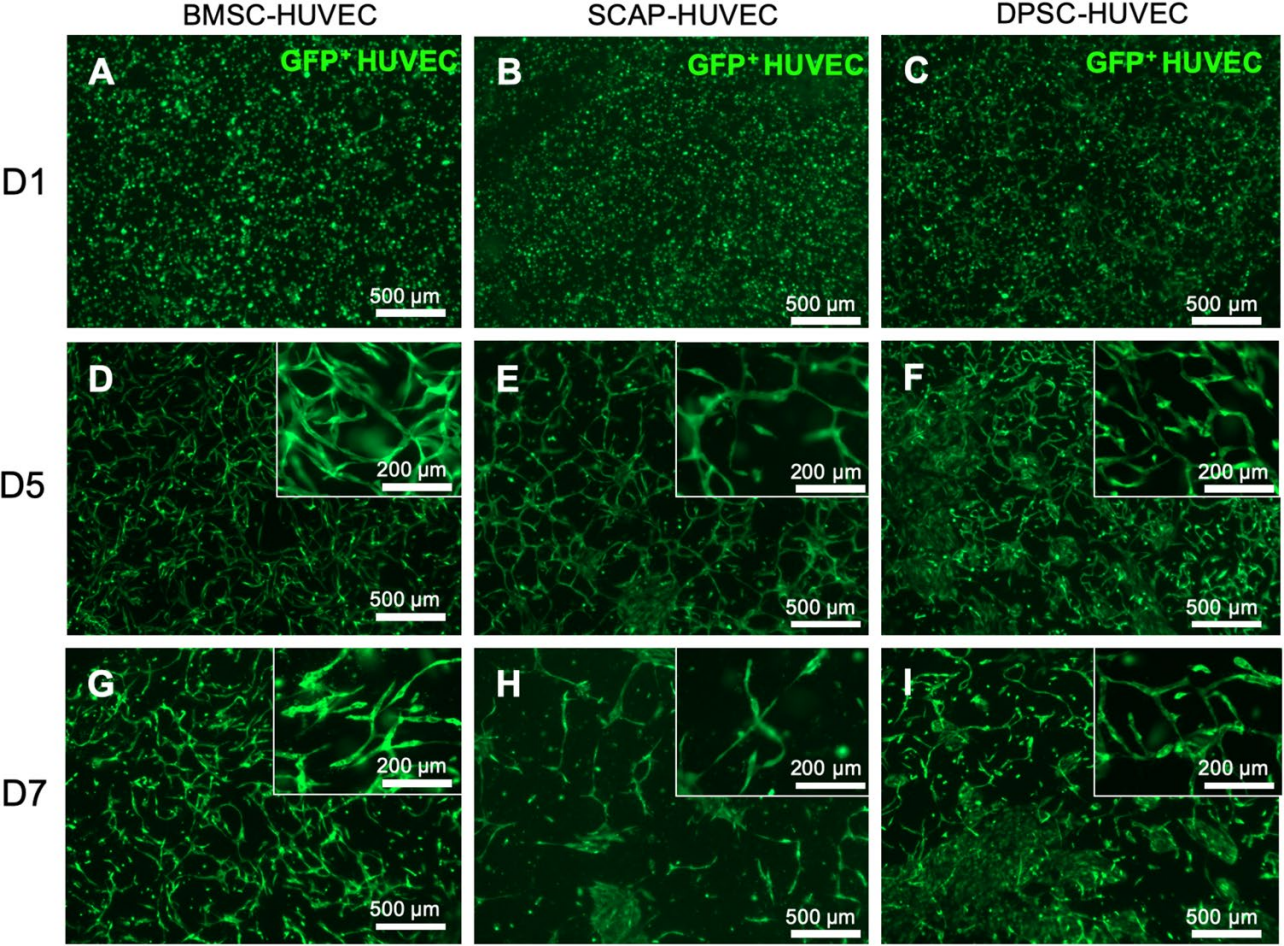
(**A**, **D**, **G**) DPSC-HUVECs, (**B**, **E**, **H**) BMSC-HUVECs, and (**C**, **F**, **I**) SCAP-HUVECs formed vascular networks. BMSC-HUVECs formed dense vascular networks by day 5 (**E**). After the pericyte formation, BMSC system remained the same (**F**), however, the DPSC (**G**) and SCAP (**I**) showed cell island formation along with vascular networks.

The main goal of Fig. 3 was to demonstrate the efficacy of the differentiation of the craniofacial tissue-derived stem cells into pericytes, in conditions that may more closely approximate clinical applications. On that note, important results have recently demonstrated the regeneration of vascularized dental pulp, alveolar bone and periodontal tissue using cell-laden hydrogels co-cultured with different lineages of endothelial and stem cells^29–32^, such as the ones used here. However, it remains unclear whether the cross comparison of different stem cells can result in better differentiation and vasculogenic ability. Figure 3 shows the formation of microvascular networks after days 5 and 7 of culture. All stem cell types formed vascular networks around day 5, and continued to stabilize around day 7. A marked feature that stood out for DPSC and SCAP were the presence of cluster of cells along with the vascular networks.

**Figure 4:**
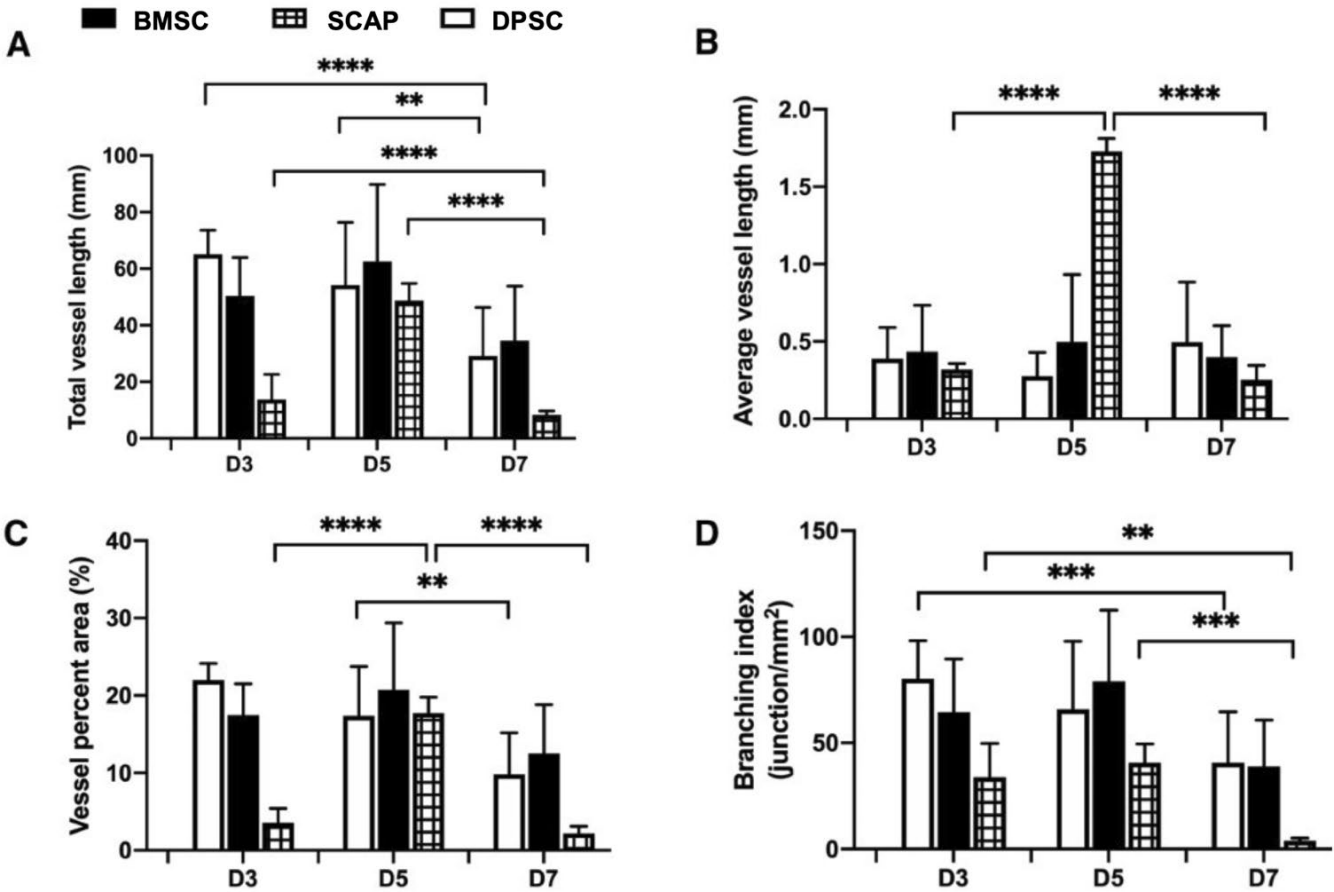
Vascular network quantification of the encapsulated stem cells-HUVECs in GelMA hydrogel. The vascular network parameters, namely, (**A**) total vessel length, (**B**) average vessel length, (**C**) vessel percent area, and (**D**) branching index showed a marked decrease from day 5 to day 7 owing to the effect of pericytes.

This feature was markedly missing in BMSC-laden samples. Regarding the specific vascular network parameters (Fig. 4), the average vessel length was higher for SCAPs on day 5, however, it drastically decreased by sevenfold on day 7 (*p* < 0.0001). The average vessel length of DPSC and BMSCs remained statistically unchanged over the 7 day period. The total vessel length of SCAP showed the same trend, as it dropped sixfold from day 5 to day 7 (*p* < 0.0001). The total vessel length of DPSC and BMSC groups also decreased 1.8-fold from day 5 to day 7 (*p* < 0.002 and *p* < 0.0002 respectively). The vessel percent area is a parameter which measures the total area covered by the vessels divided by the total image area. The vessel coverage for SCAP system decreased eightfold from day 5 to day 7 (*p* < 0.0001). This effectively signals the role of pericytes in the decrease of the coverage area. Similarly, the branching index of SCAP on day 7 decreased approximately tenfold when compared to day 5 (*p* < 0.0002). All these vascular parameters denote one common thing, a considerable reduction of vascular network parameters on day 7.

**Figure 5:**
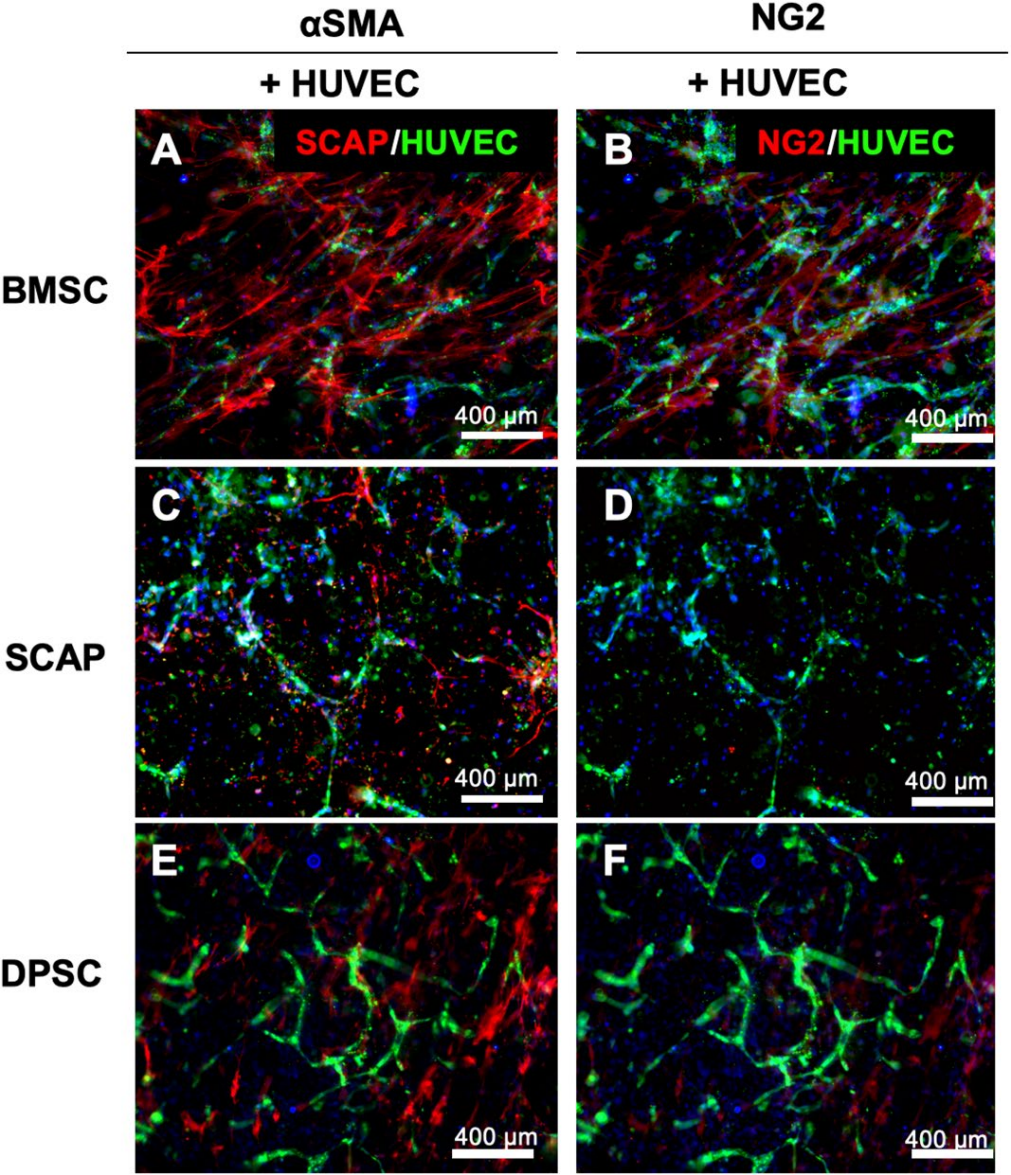
Early marker (NG2) and late marker (α-SMA) staining of differentiated stem cells. While BMSC showed the distribution of both cells (**A**, **B**), the DPSC (**C**, **D**) and SCAP (**E**, **F**) showed marked absence of NG2 positive cells.

It is well known that upon differentiation, stem cells have a tendency to line along the vasculature by extending their processes^22^. With respect to the stem cells, SCAP showed a considerable reduction in all vasculature parameters on day 7. From these results, it can be concluded that SCAP cells forms a less attractive stem cell source to engineer mature vascular systems when cultured in vitro than other stem cells. Figure 5 shows the immunofluorescence staining for α-SMA and NG2 after seven days of culture.

**Figure 6:**
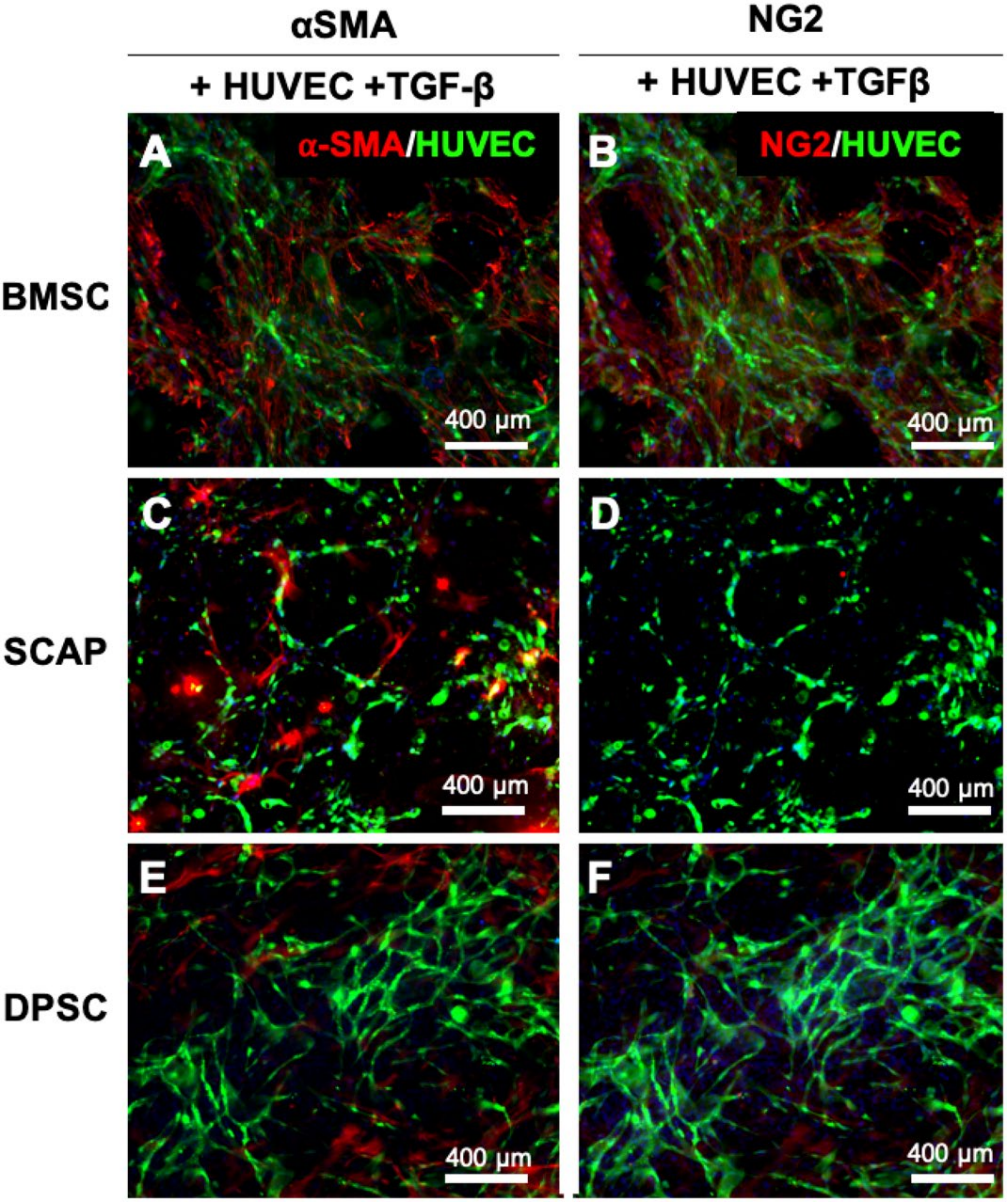
Early marker (NG2) and late marker (α-SMA) staining of differentiated stem cells with pericyte inducer TGF-β. The effect was identical with that of stem cell system involving no TGF-β. The BMSC showed the distribution of both cells (**A**, **B**), while DPSC (**C**, **D**) and SCAP (**E**, **F**) showed marked absence of NG2 positive cells.

As expected, α-SMA was highly expressed in differentiated BMSCs followed by DPSCs and SCAP. Another marker, NG2 showed a similar trend, but very less cells showed NG2 expression for DPSC and SCAPs. For SCAPs, there was hardly any NG2-expressing cells, which showed expression of NG2 is vastly different among stem cells of different source (Fig. 6).

**Figure 7:**
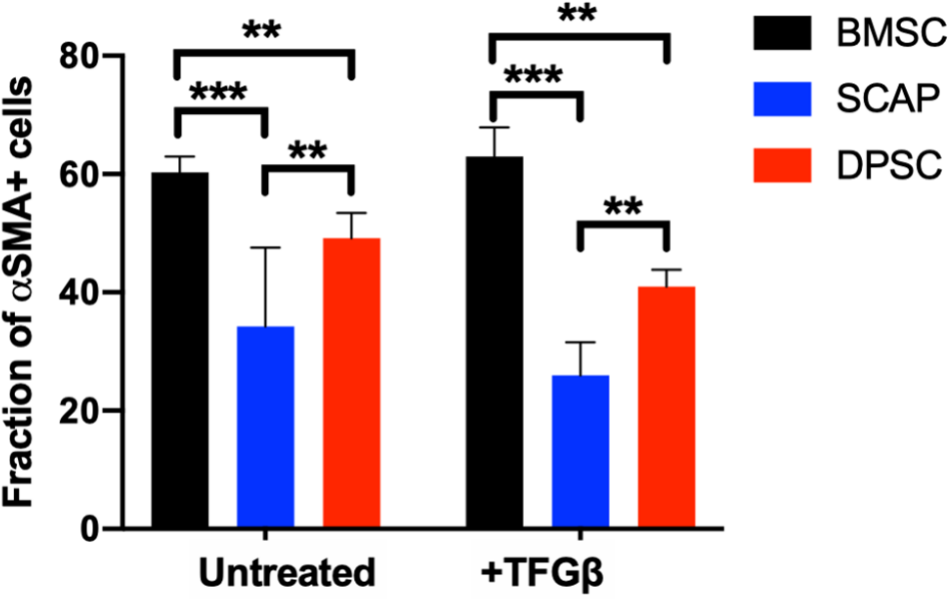
Quantification of late marker (α-SMA) staining of differentiated stem cells with and without pericyte inducer TGF-β. The effect was similar with that of stem cell system involving no TGF-β. BMSC expressed more (α-SMA), followed by DPSC and SCAP (two-way ANOVA, post hoc Tukey, *p* > 0.05).

Because ⍺-SMA was the most expressed marker for differentiated cells, we quantified the number of using ⍺-SMA positive cells in all groups using ImageJ. A fraction of 60% of BMSC expressed ⍺-SMA when co-cultured with HUVECs, while for DPSC, around 45% of cells were positive for this marker. SCAP had the least expression of ⍺-SMA with only 30% of cells being positive for the marker (Fig. 7).

Interestingly TGF-β supplementation of the cell medium did not seem to promote an increased expression of ⍺-SMA (two-way ANOVA, post hoc Tukey, *p* > 0.05).

## Discussion

The primary goal of this study was to determine the ability of different stem cells from tissues in the craniofacial region (BMSC, DPSC, and SCAP) to differentiate into pericytes and form robust microvascular capillary networks in engineered hydrogels. Our results suggest that BMSCs are significantly more potent in promoting the formation of microvascular capillaries in co-cultures with HUVECs than DPSCs or SCAP. Also, BMSCs appeared more prone to expressing higher levels of pericyte-related markers, such as α-SMA and NG2 when establishing microvascular capillaries in cell-laden hydrogels.

The most suitable cell sources for tissue engineering are cells that can be easily harvested, have high proliferative rates, are nonimmunogenic, and possess multiple-lineage differentiation capacity^33^. Therefore, stem cells have long been proposed as a desirable alternative to engineer mature vascular capillaries together with endothelial cells. Several reports have demonstrated the use of BMSCs to engineer vascular capillaries, including a few of our own^29,33–35^. Similarly, stem cells from various other sources, such as adipose-derived, induced pluripotent, embryonic, neural stem cells have also been tested for their pericyte differentiation ability^25^. However, direct comparisons between stem cells from easily accessible tissues, such as the bone marrow and extracted teeth, have remained elusive in the literature. Moreover, the use of craniofacial derived stem cells has been proposed as a desirable alternative for specific applications in regenerative dentistry and craniofacial reconstruction, and to date, information on improved candidates for craniofacial vascular engineering have remained limited^27,28,36,37^. Pericytes play a vital role in the maturation and functionality of microvascular networks by maintaining the integrity of cell–cell junction, and synthesis of the extracellular matrix (ECM) of the basement membrane^38^. Hence, the need to integrate pericytes in engineered vasculature is vital as it closely mimics the physiological functions^39^. For our study, we used early-passage cells (P4-8) considering the “stemness” window accepted by the literature. SCAP can be expanded up to 20 passages still keeping expression of stem cell markers CD73, CD90 and CD105^60^. Conversely, BMSC largely lose their in vitro differentiation capability around the 6th passage^61^ and DPSCs from passage 9 on show a decrease in their proliferative capacity and upregulation of genes such as alkaline phosphatase, dentin sialophosphoprotein and osteopontin, related to differentiation into a mineralizing cell phenotype^62^. Interestingly, we observed that although SCAP could keep their stemness for longer, their potential to differentiate into pericytes was lower than that of DPSC.

The first observation that requires attention in the comparison of stem cells alone versus co-cultures of stem cells and endothelial cells is that HUVECs appeared to be critically required to induce the differentiation of all three stem cell types in pericytes when seeded in 2D substrates, despite the use of the exact same medium conditions. This is a relevant observation, as it alludes to the ability of paracrine signals secreted from ECs to promote pericyte differentiation despite the absence of exogenous growth factors, which is typical in the field of regenerative medicine^40,41^. Cell–cell contact between BMSCs and endothelial cells has been shown to induce pericyte-like cell differentiation with upregulation of pericyte markers like CD146, NG2, and α-SMA^42^. Transforming growth factor-beta (TGF-β) and Notch signaling mediate stem cell differentiation to pericytes and smooth muscle cells^43^. In particular, the TGF-β family has been involved in regulating vascular development and barrier function^44^. When cultured, HUVECs will produce growth factors such as TGF-β and basic fibroblast growth factor^45^. To gain further insights into this particular phenomenon, we performed additional experiments using TGF-β (a commonly reported inducer of pericyte differentiation in the literature) in our medium as a positive control, and observed comparable results to those obtained with a co-culture of HUVECs and stem cells without any additives (Fig. 6). Upon the observation that all three stem cell types were capable of inducing the expression of a pericyte-related marker (α-SMA) without using TGF-β in the medium, we engineered hydrogels using a dental curing light, which we have demonstrated to be compatible with engineering of dental pulp-like tissue^46^ and vascular capillaries directly in root canals^47^. Our results demonstrated that similar to our 2D cell culture screening, BMSC were visually more capable of stimulating well-established microvascular capillary networks (Fig. 3). A similar observation was made with the 3D cell culture, where a reduction in vascular parameters after 5 days of culture was seen (Fig. 4). Usually, pericyte differentiation takes approximately 5 days, so the reduction in the average vessel length can be attributed to effect of conjoining of pericytes to capillaries^40,41,48^. The decrease in vasculature parameters can be attributed to the constrictory nature of the pericytes. α-smooth muscle actin (α-SMA) is conventionally used by pericytes to constrict capillaries^49,50^. Given that all the differentiated stem cells extensively express α-SMA, this effect may lead to constriction leading to the vessel shrinkage length and area^51^. This explains the overall behavior of the decrease in vasculature parameters in some of the stem cell we utilized. Of note, not only BMSCs formed vascular capillaries sooner than SCAP, but also, they appeared to resist against vascular regress more clearly than DPSCs, which were quantitatively comparable to BMSCs in the quantification of vasculature formation.

The main challenge while dealing with identification of pericytes is the lack of a single definitive marker to identify pericytes. NG2, Desmin, α-SMA, and PDGFRβ are some of the markers used in the field to identify pericytes^52^. NG2 chondroitin sulfate proteoglycan is a structurally unique, integral membrane proteoglycan expressed in several types of immature cells, and by mural cells during vascular morphogenesis^53^. Therefore, to further confirm the differentiation of stem cells into pericytes and elucidate the specificity of the perivascular phenotype adopted by these cells, in addition to α-SMA, we also characterized the expression of NG2 for cell co-cultured with HUVECs in our engineered hydrogels. Of note, NG2 also represents the early stage marker of pericytes, while α-SMA is considered a late stage protein in the differentiation process^40,54^. Thus, these markers may not only indicate the maturity of the pericyte cells but also can give an idea about the phenotype which the pericytes can take in the microvascular tree. Moreover, it is well known that pericytes adopt different morphologies along the microvascular bed, and similar to their morphologies, the expression of pericyte markers also continues to vary^55^. For instance, NG2 is highly expressed in capillary pericytes, while it continues to decrease for pericytes on arterioles and venules^54^. On the contrary, α-SMA is expressed less in the capillary pericytes, but its expression increases for pericytes on arterioles and venules. In fact, with NG2 and α-SMA expression, one can distinguish the three subsets of human pericytes in the following way: capillaries (NG2 +  α-SMA-), venules (NG2-α-SMA +) and arterioles (NG2 + α-SMA +)^14,56,57^. In our results, both α-SMA and NG2 were expressed in BMSCs, whereas DPSC showed a higher expression of α-SMA positive cells than NG2 positive cells, and SCAP hardly showed any NG2 positive cells despite the consistent expression of α-SMA. Therefore, it appears as though BMSC can more easily adopt diverse pericyte-related phenotypes that are associated with mural cells covering of arterioles, capillaries, and venules, whereas DPSCs and SCAP may be slightly more tilted towards mural cells covering the arterioles and venules, and less on capillaries. This difference is possibly due to the location from which the stem cells are isolated. SCAPs are isolated from the root of a developing immature teeth, while the DPSCs are extracted from the inner tooth pulp of adult molars^28,58^. Further, it is well known that vasculature density differs in the root (apical area) and crown parts of the pulp^59^. Therefore, this might be the reason for the varied levels of marker expression among the stem cells.

In summary, our results point to important observations regarding the ability of stem cells from different craniofacial tissues to differentiate into pericytes and form microvascular capillary networks in engineered hydrogels. Accordingly, BMSC appear more suitable for engineering of pericyte-supported capillaries than DPSCs or SCAPs. We propose that these results may be useful to facilitate the repair and regeneration of craniofacial and dental tissues.

## Methods

### Cell culture

All experiments used green fluorescent protein (GFP)-expressing human umbilical vein endothelial cells (HUVECs—cAP0001GFP, Angioproteomie) (P5-10), which were cultured in Endothelial Growth Medium (EGM) (cAP-02, Angioproteomie) on gelatin (0.1% (w/v)) coated substrates. SCAP (P4-8) (primary cells donated by Dr. Diogenes, University of Texas), and DPSC (P4-8) (primary cells, catalog # PT-5025, Lonza, Basel, Switzerland) were cultured in alpha-MEM supplemented with L-glutamine, 10% (v/v) fetal bovine serum and 1% (v/v) penicillin–streptomycin. Primary cultures of bone marrow human mesenchymal stem cells (BMSC) (donated by Dr. Brian Johnstone, OHSU Orthopedics) on passages 3 and 4 were cultured in alpha-MEM supplemented with L-glutamine, 10% (v/v) fetal bovine serum and 1% (v/v) penicillin–streptomycin. All cells were maintained in an incubator at 37 °C, 5% CO_2_, with media replacement every 2 days.

### Sample preparation

GelMA hydrogel precursor was synthesized by reacting gelatin with methacrylic anhydride according to previously established protocols^60^. GelMA hydrogels for 2D and 3D models were separately formulated to achieve the material properties that support endothelial monolayer formation and microcapillary network formation respectively^29,46,61^. Consequently, for the 2D assays, we used 10% (w/v) GelMA precursor, 0.05% (w/v) LAP in DPBS^62^, whereas the hydrogels for 3D experiments were formulated with 5% (w/v) GelMA precursor with 0.075% (w/v) LAP in DPBS^63^. GelMA hydrogel samples (9.2 mm diameter, 150 micron thick) were fabricated by dispensing 10 µl of the appropriate GelMA formulation on TMSPMA coated glass between 150 micron thick spacers and photopolymerizing with a dental curing light (Valo) for 5 s. For the pericyte differentiation assays in 2D, each stem cell line was seeded on to the surface of the hydrogel either alone or in co-culture with GFP-HUVECs (1:4) at a density of 75 cells/mm^2^ for 7 days in modified EGM. In order to fabricate 3D cell-laden constructs, a co-culture of GFP-HUVECs and stem cells were suspended in the hydrogel precursor in a 4:1 ratio at a density of 5 × 10^6^ cells/ml prior to fabrication. All samples were cultured for a 7 day period. For positive control, 2 ng/mL TGF-β was added to the modified EGM medium.

### Immunostaining

Prior to immunostaining, samples were fixed with 10% (v/v) formaldehyde and permeabilized using 0.1% (v/v) Triton X-100. They were then blocked with 1.5% (w/v) bovine serum albumin (Sigma Aldrich) in DPBS for 1 h, following which they were treated with Image-iT FX signal enhancer (Invitrogen, CA) for 30 min. Samples were then immunostained with antibodies against α SMA (0.4 µg/ml) (Thermofisher Scientific, # MA5-11547) and NG2 (R&D systems, #FAB25851R) and imaged using fluorescence microscopy. As a measure of the expression of α-SMA, the intensity of fluorescence signal from the antibody against α-SMA was quantified for all the cells in a sample using Image J software. The cells with fluorescent signal above that of the background were identified as α-SMA positive cells.

### Vascular network quantification

Angiotool (NIH) software in Fiji (ImageJ, NIH) was used to quantify the vasculature formed in the cell-laden hydrogels. The fluorescence images of the GFP-HUVEC networks were processed through a sequence of steps including image segmentation, skeletonization, optimal thresholds for vessel diameter and signal strength, and removal of small particles. From this, various vessel morphometry parameters such as vessel percent area, total vessel length, average vessel length, and vascular branching index were calculated^29^.

### Statistical analysis

All assays were performed for n = 3 replicates and data presented as mean ± standard deviation. Statistical analysis was conducted using two-way ANOVA followed by Tukey post hoc tests (a = 0.05) with Graphpad Prism 8.
